# Ripple AT Plus — isthmus-guided vs conventional ablation in the treatment of scar-related atrial tachycardia: study protocol for a randomised controlled trial

**DOI:** 10.1007/s10840-023-01607-8

**Published:** 2023-08-18

**Authors:** Balrik Kailey, Ian Kemp, Martika Taylor, Jennifer Crooks, George Katritsis, Michael Koa-Wing, Shahnaz Jamil-Copley, Nick Linton, Prapa Kanagaratnam, Dhiraj Gupta, Vishal Luther

**Affiliations:** 1 Liverpool Heart & Chest Hospital, Thomas Drive, Liverpool, L14 3PE UK; 2 Imperial College London, Imperial College Healthcare NHS Trust, London, UK; 3 Nottingham University Hospital, Nottingham, UK

**Keywords:** Atrial tachycardia, Linear ablation, Isthmus, Catheter ablation

## Abstract

**Background:**

Catheter ablation is routinely used to treat scar-related atrial tachycardia (s-AT). Conventional ablation often involves creating anatomical “lines” that transect myocardial tissue supporting reentry. This can be extensive, creating iatrogenic scar as a nidus for future reentry, and may account for arrhythmia recurrence. High-density mapping may identify “narrower isthmuses” requiring less ablation, with ripple mapping proven to be an effective approach in identifying. This trial explores whether ablation of narrower isthmuses in s-AT, defined using ripple mapping, results in greater freedom from arrhythmia recurrence compared to conventional ablation.

**Methods:**

The Ripple-AT-Plus trial (registration ClinicalTrials.gov, NCT03915691) is a prospective, multicentre, single-blinded, randomised controlled trial with 12-month follow-up. Two hundred s-AT patients will be randomised in a 1:1 fashion to either “ripple mapping-guided isthmus ablation” vs conventional ablation on the CARTO3 ConfiDENSE system (Biosense Webster). The primary outcome will compare recurrence of any atrial arrhythmia. Multicentre data will be analysed over a secure web-based cloud-storage and analysis software (CARTONET^TM^).

**Conclusion:**

This is the first trial that considers long-term patient outcomes post s-AT ablation, and whether targeting narrower isthmuses in the era of high density is optimal.

## Background

Scar-related atrial tachycardia (s-AT) can cause significant patient symptoms. These arrhythmias are often iatrogenic, secondary to scar created from prior atrial fibrillation (AF) ablation or cardiac surgery [1, 2]. As AF ablation is ever increasing, so too is the burden of these arrhythmias on healthcare systems [3, 4]. 3D mapping and catheter ablation is proven superior to anti-arrhythmic drugs in the treatment of these arrhythmias [5, 6]. However, arrhythmia recurrence remains a real issue for electrophysiologists.

S-AT ablation often involves creating “lines” that transect atrial tissue that support reentry. These lines are classified by their bordering anatomical structures (e.g. roof line, mitral line). Blocking conduction across these lines is important to prevent tachycardia recurrence. These lines can be significant in length, and achieving block can be challenging. Arrhythmia recurrence can occur secondary to “gaps” within these lines. Furthermore, if the delivery of ablation is excessive, this can cause iatrogenic scar and future reentry.

In the era of high-density mapping, “narrower isthmuses” supporting slow conduction bounded by scar are considered alternative ablation targets compared to linear anatomical based lesions. A comparison of outcomes between ablation targeting a narrow isthmus or anatomical linear lesions has yet to be performed; hence, operators may vary in their preferred ablation strategy. We hypothesise that an isthmus-targeted ablation of s-AT is superior to conventional ablation in reducing long-term recurrence, primarily as it may require less ablation with shorter procedural duration.

To test this hypothesis, we plan to identify narrow isthmuses using the “Ripple Map scar thresholding” technique, as we have shown this to be superior to conventional mapping in identifying narrow isthmuses (Ripple-AT Study) [7]. We look to compare outcomes of S-AT ablation targeting the narrowest identified isthmus with ripple mapping, with conventional mapping and ablation approaches (which often require linear lesions).

## Methods

### Study design

Ripple-AT Plus (ClinicalTrials.gov identifier number NCT03915691) is planned as a prospective, multicentre, single-blinded, randomised controlled superiority trial. The protocol was approved by the research ethics committee 19/LO/0637.

The target recruitment number is 200 with a 1:1 randomisation to either a “Ripple Map scar thresholding”-guided isthmus ablation or a “conventional mapping and ablation”. The primary end-point is documented ECG or Holter recurrence of any atrial arrhythmia which will be assessed at 3, 6, 9 and 12 months. All patients will undergo a 24-h Holter at 12 months.

### Randomisation

Randomisation will be done prior to the catheter ablation procedure. This will be done via a secure web interface with treatment allocation stratified by centre and controlled using a block system with random variation.

The study will be performed according to the study protocol, Good Clinical Practice as defined by the National Institute of Health Research (NIHR) and all applicable regulatory requirements.

### Ablation analysis

Anonymised mapping and ablation data will be analysed by the research team using a novel cloud-based storage and analysis software (CARTONET Microsoft Azure cloud, Biosense Webster) that securely transfers and uploads 3D mapping cases within the CARTO-3 system for remote review (https://eu.cartonet.net/login) via a Siemens teamplay gateway (Siemens, Malvern, PA).

### Eligibility criteria

Potentially eligible patients will be those referred for catheter ablation of AT by their direct care team, according to standard clinical guidelines. The investigators will identify potentially eligible patients for recruitment according to pre-defined inclusion and exclusion criteria.

### Inclusion

1. Referred for catheter ablation of s-AT (post-surgical*, post-ablation, idiopathic atypical atrial flutter**) by the direct clinical care team.
2. Male or female, aged ≥ 18 years old.
3. Able to consent for recruitment to the trial and the catheter ablation procedure.

*Post-surgical refers to cardiac surgery specifically and not CABG alone.

**Idiopathic atypical flutter includes any non-CTI flutter dependent upon constrained activation/slow conduction involving idiopathic scar to facilitate re-entry.

### Exclusion

1. Contraindication to catheter ablation as deemed by the clinical team.
2. Typical atrial flutter or atrial fibrillation on ECG.

Patients not in stable AT for the duration of mapping, or those with non-inducible AT on the day of the procedure, will be excluded from the study.

### Procedural setup

The peri-procedural anticoagulation regime is according to physician discretion but in line with established HRS/ESC guidelines, and TOE or CT may be used to exclude LA or LAA thrombus. General anaesthesia or sedation will be used as per local preference. 3D electroanatomical mapping will be performed using CARTO with the ConfiDENSE and ripple mapping modules (Biosense Webster Inc.). Once femoral vein access is established, a multi-polar electrode catheter will be inserted into the coronary sinus (CS) as a reference. The direction of activation on the CS catheter will be assessed. Following determination of the chamber of interest, high-density mapping of that chamber or both chambers is undertaken.

Intracardiac mapping will be performed in a stable AT using a multipolar catheter (Pentaray or Octaray) to cover the anatomy with colour threshold of 5. Standard ConfiDENCE filters will be applied to avoid erroneous point collection on the map. These include: (1) cycle length stability within 5% range; (2) electrode position stability within 2mm; (3) LAT stability filter at 3 milliseconds; (4) tissue proximity index to the endocardial surface.

### Interventions

#### Arm 1

**Fig. 1:**
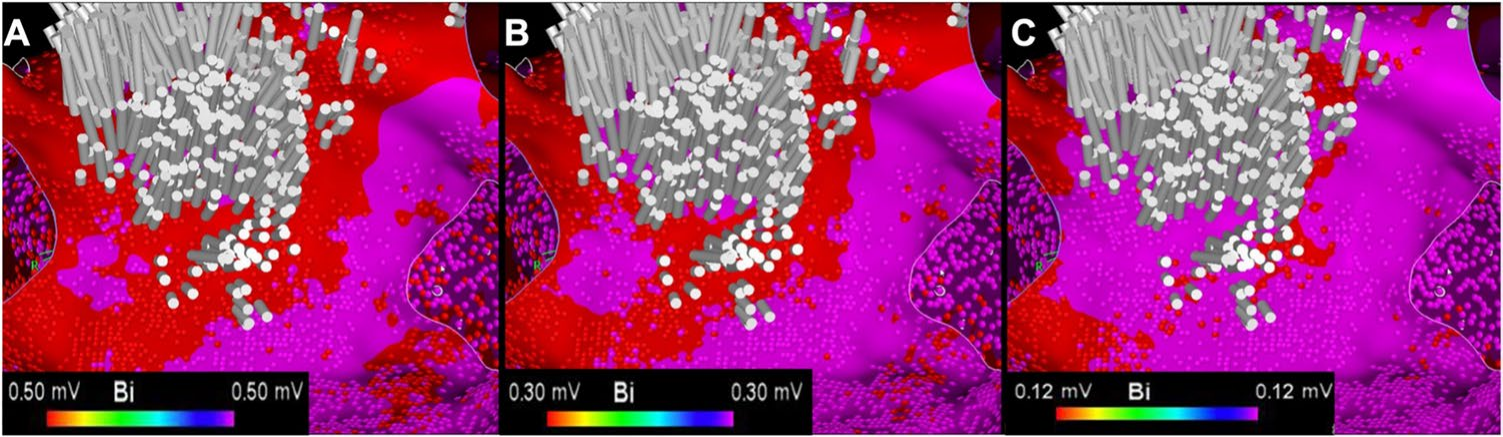
Ripple Map scar thresholding technique. **A** The Ripple Map of this s-AT circuit is played on a bipolar voltage map set at 0.50–0.50 mV. Ripple Bars are seen in the tissue coloured red (< 0.50mV). **B** The voltage limits are reduced sequentially to 0.30–0.30mV. Ripple Bars can still be seen in the tissue coloured red. **C** The voltage limits are further reduced until no Ripple Bars are seen in the tissue coloured red. This defines the scar threshold at 0.12 mV

**Fig. 2:**
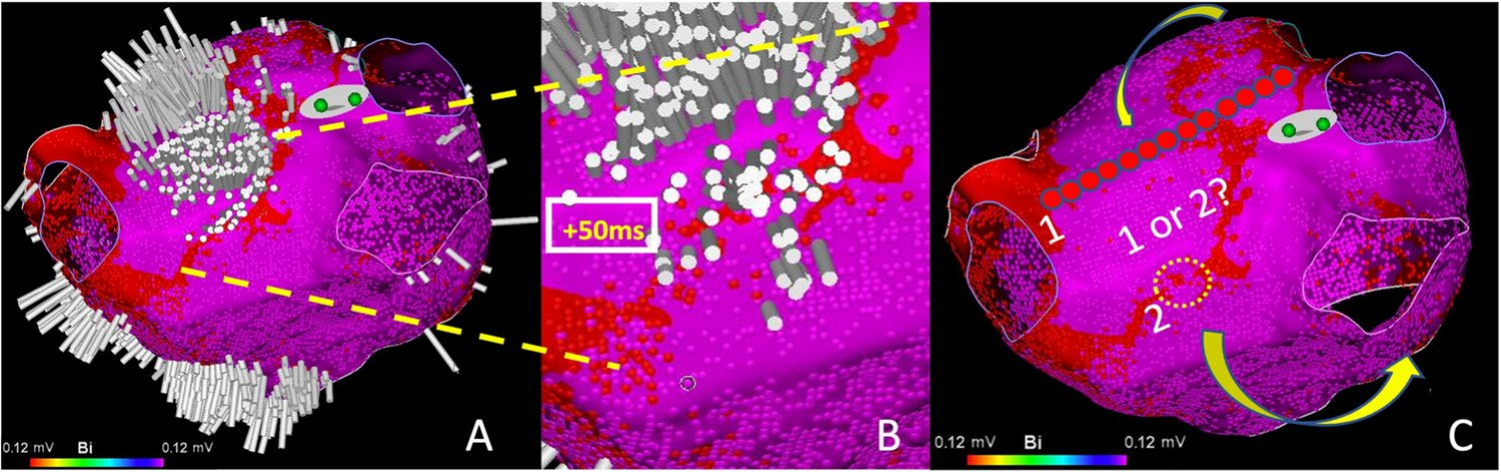
Post AF ablation scar related Atrial Tachycardia (s-AT). (**A**) Ripple activation travels craniocaudal along the left atrial roof. The Ripple Scar Thresholding technique defines the scar threshold at 0.12mV. (**B**) A narrow isthmus bordered by scar is revealed on the anterior wall (activation time +50ms). (**C**) The two potential ablation strategies are a conventional roof line (1) or narrow isthmus ablation (2)

Ripple mapping displays acquired EGMs as a white oscillating bar which can be displayed over any base map. This property allows operators to view a Ripple Map over a bipolar voltage map on the same display. This allows the operator to identify areas of slow conduction in low-voltage regions. Cases will be commenced with bipolar scar thresholds of 0.5 mV. Operators will identify areas of Ripple activation, and the voltage settings will be sequentially reduced until a threshold is reached where no further Ripple Bar activation is seen. This scar thresholding method is shown in Fig. 1. The operators are then encouraged to identify the narrowest isthmus for ablation (Fig. 2). Entrainment is permitted after mapping if the operator feels it is necessary for the diagnosis.

#### Arm 2

In the conventional mapping and ablation arm, all conventional CARTO activation mapping techniques (including LAT mapping, Extended Early Meets Late, Coherence) can be utilised as per operator preference. Entrainment is permitted after mapping if the operator feels it is necessary to make a diagnosis. Ripple mapping is not permitted. Once a diagnosis is made, the ablation set is planned as per operator preference.

### Ablation

Radiofrequency ablation is delivered by the SmartTouch ThermoCool Catheter/Q-Dot catheter and its appropriate generator (Biosense Webster Inc.). Power and duration of radiofrequency energy delivered will be standardised using modified CLOSE protocol [8]. Each ablation lesion will be delivered using an inter-lesion distance of < 6 mm targeting an ablation index no greater than 400 on the posterior wall and 550 on the anterior wall or an impedance drop of 10 ohms. Standardising ablation delivery allows data collection regarding overall ablation by counting the number of lesions and also the length of the ablation lesions.

If the tachycardia circuit changes after ablation, then the protocol above as per the allocated arm is followed. If ablation is unsuccessful (defined by no restoration of sinus rhythm), then further mapping (as per the allocated arm) and ablation is permitted. Once sinus rhythm has been restored, all ablation sets including prior lesions (e.g. previous PVI) are checked using the allocated mapping technique to ensure they are still blocked with further ablation permitted if reconnected. In those with a documented history of AF, a de novo bilateral WACA is permitted at this stage if not already done as part of planned lesion set. If sinus rhythm is not restored at the end of the case, patients undergo DCCV to sinus rhythm. The use of AAD’s is documented throughout the study. Clinicians are permitted to adjust the AAD therapy as appropriate. The study does not seek to influence this practice.

### Blinding

The patient, the local clinical team assessing the patient during the follow-up period and the research team assessing endpoints will all be blinded to which arm the patient was assigned to.

### Study endpoints

**Fig. 3:**
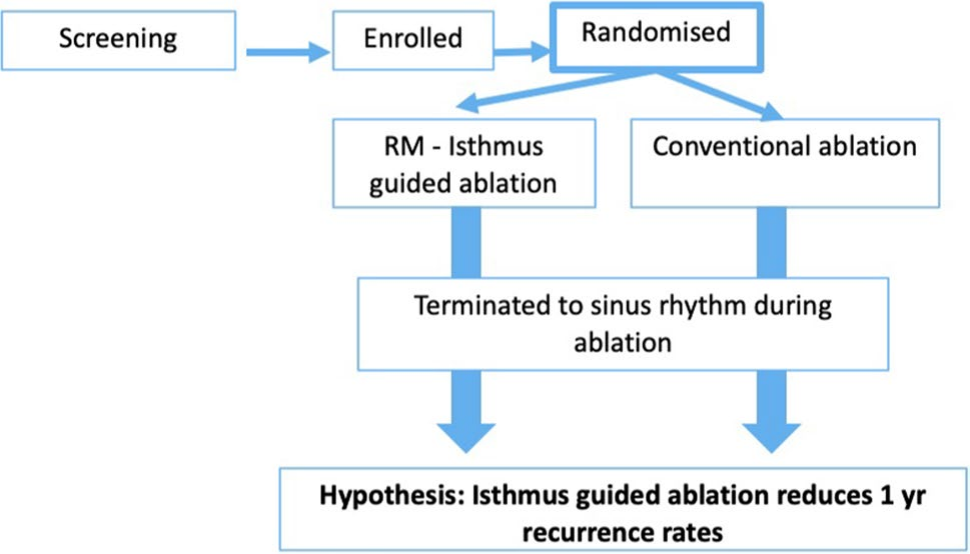
Ripple AT Plus study workflow

The primary study endpoint is an episode of sustained atrial arrhythmia (Fig. 3) occurring at any time in the 12-month period after catheter ablation. There will be a 2-month blanking period after ablation in which atrial arrhythmias are not considered. Endpoints are assessed at 3, 6, 9 and 12 months. An atrial arrhythmia (atrial fibrillation, atrial flutter, atrial tachycardia) is defined (> 30 s) on a Holter Monitor or documented on a single 12 lead ECG [9]. As patients with AT recurrence may degenerate to Afib at the time of ECG, it was included as a primary endpoint. The date of recurrence for each atrial arrhythmia will be recorded. In addition to any ECG and Holter Monitors performed by the clinical team, all patients will undergo an additional 24-h Holter at 12 months.

### Secondary endpoints

Other secondary endpoints measured are:

- Acute procedural success (defined by restoration of sinus rhythm through ablation without need for DCCV)
- Procedure time
- Ablation delivered (lesion number and length of ablation set)

### Endpoint adjudication

Primary endpoints will be assessed at 3-, 6-, 9- and 12-month intervals by a blinded researcher. They will review all clinical data (including Holter’s and ECG’s) for evidence of atrial arrhythmia, as well as recording the results of a further 24-h Holter at 12 months. Endpoint adjudication will be done by an independent Adjudication Committee (EAC). The EAC will centrally review all recurrences to ensure they have been classified correctly.

### Sample size calculation

The sample size for this study is based on previous evidence published by our group. In a non-randomised, prospective study comparing ripple mapping–guided ablation to conventional mapping–guided ablation, the diagnostic accuracy was 90% and 65% respectively [10]. A similar effect size was also seen in a randomised prospective study (Ripple-AT Study), showing acute success was 90.5% and 70.7% respectively. In order to detect a difference between arms with 90% power and an alpha value of 0.05, at least 80 patients would be needed to be randomised to each treatment arm. Accounting for potential non-completion of protocol (10%) and loss to follow-up (10%), a minimum of 200 patients will need to be enrolled. Power calculations were performed using the G*Power software (University of Dusseldorf).

### Funding and sponsorship

Ripple-AT-Plus is funded by Biosense Webster (subsidiary of Johnson&Johnson) as part of an Investigator Initiated Grant (IIS Grant-618) and is sponsored by Liverpool Heart & Chest Hospital NHS Foundation Trust.

### Progress

This study currently has 3 recruiting NHS sites with an aim of at least 10 UK centres. It commenced recruiting in October 2022 and has randomised 33 patients to date.

## Discussion

The Ripple-AT Plus Study is the first blinded randomised trial that considers long-term outcomes in s-AT ablation. It will compare ablation targeting the “narrowest isthmus” identified using the ripple mapping scar thresholding technique [11], against conventional mapping (e.g. LAT mapping) and ablation techniques, which often employs standardised anatomically defined linear lesions. At present, it is not known whether targeting a narrower isthmus leads to improved outcomes.

There has been significant progress over the last decade in high-density 3D mapping techniques. Despite this, in real-world practice, operators continue to deliver standardised anatomical lesions, which can require more extensive ablation delivery. High-density mapping allows us to appreciate narrower isthmuses, which may require less ablation [11, 12]. Curtailing the amount of ablation delivery will reduce the amount of iatrogenic scar created, with scar known to act as a nidus for future arrhythmia recurrence [13]. As ablation invariably creates new scar, we hypothesise less ablation is less pro-arrhythmic, and this will be studied in a randomised fashion. However, whether a narrow isthmus mapped in tachycardia is truly narrow (bordered by fibrosis) or bordered by refractory tissue observed at a faster atrial cycle length (i.e. functional block) is unknown. This will be analysed with remapping in sinus rhythm post ablation.

This trial follows on from the Ripple AT study from our group, which was the largest ever randomised mapping trial involving scar-related atrial tachycardia [7]. This study demonstrated that ripple mapping scar thresholding was superior to Local Activation Time mapping in guiding successful ablation. In a sub-analysis of the peri-mitral circuits later published from this study [14], almost 4/5 cases had a clear isthmus bounded by scar, < 1 cm in length and identifiable using Ripple Mapping with a 100% termination rate when targeted for ablation. In contrast, conduction block was difficult to achieve using linear ablation of the conventional posterior mitral isthmus and often required long anatomical ablation lines with more than double the amount of ablation. In this study, crossover between mapping arms was permitted; therefore, it remains unknown whether using the ripple mapping scar thresholding technique to guide ablation translates to longer term arrhythmia freedom. Follow-up data from this study (unpublished) showed a 25% recurrence, all atrial arrhythmia over 12-month follow-up showing a clear need to prioritise longer-term outcomes, instead of acute ablation success [7].

We feel the ripple mapping scar thresholding technique is the gold standard for diagnosing the “narrowest isthmus” and is robust enough to standardise in a multi-centre approach. As this software is commercially available, it can be applied worldwide.

This study may offer insight into the patient characteristics that may not respond well to ablation. Whether a certain burden of scar portends early recurrence is important in clinical selection of individuals that are referred for ablation, particularly with the acceleration of conduction system pacing and AV node ablation as an alternate treatment strategy [15, 16].

**Fig. 4:**
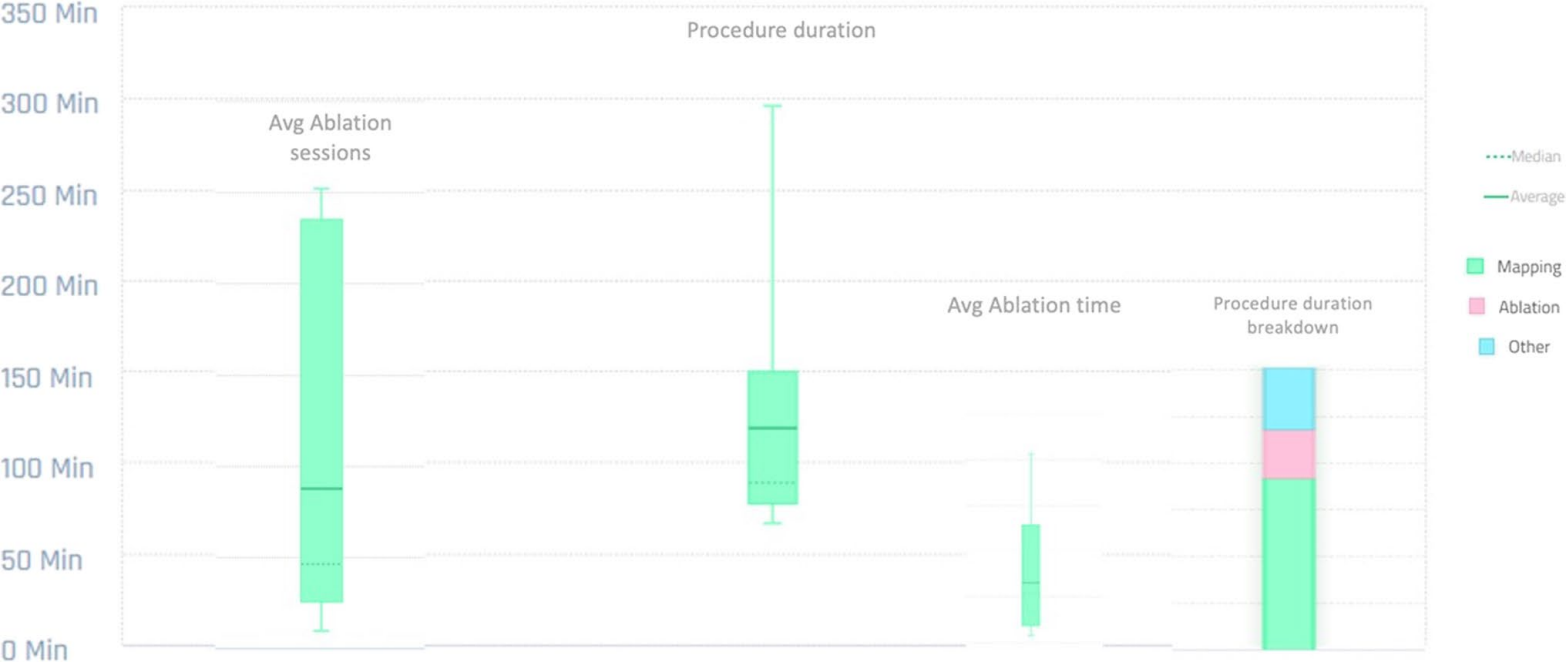
Graphical dataset presented using CARTONET™. Summarised graphical dataset for the first 8 study cases presented by CARTONET™ (including total procedural duration, mapping vs ablation times, ablation lesions)

The COVID-19 pandemic enforced long-term changes in healthcare towards remote working, implementing digital systems that support the safe online sharing of clinical information. CARTONET is a secure, simple to use and efficient cloud-based AI/machine learning system that offers access to clinically relevant data-sets from the CARTO system remotely from the hospital premises. We have already demonstrated the feasibility of this technology as a research tool in analysing the first cases recruited to the Ripple AT PLUS study from different hospitals (Fig. 4) [17]. Our study will examine its ability to improve the efficiency of multicentre clinical electrophysiology research on a larger scale.

## Limitations

This study uses standard binary clinical endpoints with ECG or Holter evidence to define recurrence as opposed to continuous monitoring.

Operator experience may bias diagnostic ability within each interventional arm, though a multicentre approach with numerous operators may mitigate against this.

This study does not seek to influence anti-arrhythmic drug usage, and it is possible that this may differ between arms.

The conventional arm the protocol is permissive allowing numerous different approaches which may make the comparator arm heterogeneous.

## Summary

This study hopes to determine whether targeting the narrowest isthmus identified using the ripple mapping scar thresholding technique in s-AT ablation leads to longer-term freedom from arrhythmia when compared to a conventional approach.
